# Lactobacillus plantarum MB452 enhances the function of the intestinal barrier by increasing the expression levels of genes involved in tight junction formation

**DOI:** 10.1186/1471-2180-10-316

**Published:** 2010-12-09

**Authors:** Rachel C Anderson, Adrian L Cookson, Warren C McNabb, Zaneta Park, Mark J McCann, William J Kelly, Nicole C Roy

**Affiliations:** 1 AgriFoods & Health Section, Food & Textiles Group, AgResearch Grasslands, Private Bag 11008, Palmerston North 4442, New Zealand; 2 Food & Textiles Group, AgResearch Grasslands, Private Bag 11008, Palmerston North 4442, New Zealand; 3 Riddet Institute, Massey University, Private Bag 11222, Palmerston North 4442, New Zealand; 4 Bioinformatics, Mathematics & Statistics Section, Applied Biotechnology Group, AgResearch Grasslands, Private Bag 11008, Palmerston North 4442, New Zealand; 5 Ruminant Nutrition & Microbiology Section, Food & Textiles Group, AgResearch Grasslands, Private Bag 11008, Palmerston North 4442, New Zealand

## Abstract

**Background:**

Intestinal barrier function is important for preserving health, as a compromised barrier allows antigen entry and can induce inflammatory diseases. Probiotic bacteria can play a role in enhancing intestinal barrier function; however, the mechanisms are not fully understood. Existing studies have focused on the ability of probiotics to prevent alterations to tight junctions in disease models, and have been restricted to a few tight junction bridging proteins. No studies have previously investigated the effect of probiotic bacteria on healthy intestinal epithelial cell genes involved in the whole tight junction signalling pathway, including those encoding for bridging, plaque and dual location tight junction proteins. Alteration of tight junction signalling in healthy humans is a potential mechanism that could lead to the strengthening of the intestinal barrier, resulting in limiting the ability of antigens to enter the body and potentially triggering undesirable immune responses.

**Results:**

The effect of *Lactobacillus plantarum *MB452 on tight junction integrity was determined by measuring trans-epithelial electrical resistance (TEER) across Caco-2 cell layers. *L. plantarum *MB452 caused a dose-dependent TEER increase across Caco-2 cell monolayers compared to control medium. Gene expression was compared in Caco-2 cells untreated or treated with *L. plantarum *MB452 for 10 hours. Caco-2 cell RNA was hybridised to human oligonucleotide arrays. Data was analysed using linear models and differently expressed genes were examined using pathway analysis tools. Nineteen tight junction-related genes had altered expression levels in response to *L. plantarum *MB452 (modified-P < 0.05, fold-change > 1.2), including those encoding occludin and its associated plaque proteins that anchor it to the cytoskeleton. *L. plantarum *MB452 also caused changes in tubulin and proteasome gene expression levels which may be linked to intestinal barrier function. Caco-2 tight junctions were visualised by fluorescent microscopy of immuno-stained occludin, zona occludens (ZO)-1, ZO-2 and cingulin. Caco-2 cells treated with *L. plantarum *MB452 had higher intensity fluorescence of each of the four tight junction proteins compared to untreated controls.

**Conclusions:**

This research indicates that enhancing the expression of genes involved in tight junction signalling is a possible mechanism by which *L. plantarum *MB452 improves intestinal barrier function.

## Background

The intestinal barrier is the largest interface between man and the external environment, and the maintenance of its integrity has an important role in preserving health. When intestinal barrier function is compromised, it can become "leaky" allowing pathogens and toxins to enter the body. The function of the intestinal barrier is compromised in human conditions such as Inflammatory Bowel Diseases (Crohn's Disease and Ulcerative Colitis) [1], Irritable Bowel Syndrome [2] and some kinds of food-borne infections [3]. Moreover, intestinal barrier function can be temporarily impaired during times of stress [4] and it inevitably deteriorates with aging [5]. In addition, increased intestinal permeability can also result in pathological changes in distant organs and tissues, which can lead to further complications in susceptible individuals such as asthma [6], chronic heart failure [7], type-1-diabetes [8], chronic fatigue syndrome [9] and depression [10].

A critical component of the intestinal barrier is the intercellular junction complexes between adjacent intestinal epithelial cells which form a semi-permeable diffusion barrier. These intercellular complexes consist of tight junctions, adherens junctions, desmosomes and gap junctions [11]. The tight junctions are the most apical and are responsible for controlling the permeability of the paracellular pathway. Tight junctions are formed by protein dimers that span the space between adjacent cell membranes. There are over 40 proteins with well recognised roles in tight junction formation. These proteins can be divided into three functional categories: 1) bridge proteins which form a web between adjacent cell membranes; 2) plaque proteins which anchor bridge proteins to the actin cytoskeleton; and 3) dual location proteins which are not continuously associated with the tight junctions and also act as transcription factors.

The maintenance or enhancement of intestinal barrier function is a beneficial property that some probiotic bacteria exert. Some probiotics have been shown to ameliorate intestinal permeability induced by pathogens *in vitro *[12,13]; whereas, others probiotic bacteria have been shown to enhance tight junction integrity between intestinal epithelial cells that are not weakened [13-15]. Existing mechanistic studies have focused on the ability of probiotics to prevent alterations to few tight junction bridging proteins in disease models, e.g. the effect of VSL#3 on dextran sodium sulphate-induced colitis in mice [16] and the effect of *Lactobacillus plantarum *CGMCC 1258 on Enteroinvasive *E. coli *ATCC 43893 (serotype O124:NM)-induced barrier disruption *in vitro *[17]. The effect of probiotics on tight junction proteins in a healthy intestinal barrier have not been reported, nor the effect of probiotic bacteria on epithelial cell genes involved in the whole tight junction signalling pathway, including those encoding for bridging, plaque and dual location tight junction proteins. Alteration of tight junction signalling in healthy humans is a potential mechanism that could lead to the strengthening of the intestinal barrier, resulting in limiting the ability of antigens to enter the body and potentially triggering undesirable immune responses.

The hypothesis of this research was that probiotic bacteria that increase intestinal barrier function achieve this, partly, by increasing the expression of the genes involved in tight junction signalling in healthy intestinal epithelial cells. *L. plantarum *MB452 isolated from the probiotic product VSL#3 was chosen as the test bacterium because it has a robust, repeatable, positive effect tight junction integrity, as measured by the trans-epithelial electrical resistance (TEER) *in vitro *(unpublished results). VLS#3, which is a mixture of eight bacteria including *L. plantarum *MB452, has previously been reported to enhance tight junction integrity *in vitro *[18], reduce colitis in rodent models [19,20] and improve human intestinal health [21-23]. The effect of *L. plantarum *MB452 on intestinal epithelial cells was investigated *in vitro *using human colon cancer cells (Caco-2 cells), a commonly used model of the intestinal epithelium that spontaneously form tight junctions between adjacent cells, and trans-epithelial electrical resistance assays, whole genome microarray analysis, and fluorescent microscopy of tight junction proteins.

## Results

### Effect of *L. plantarum *MB452 on TEER was dose-dependent

**Figure 1:**
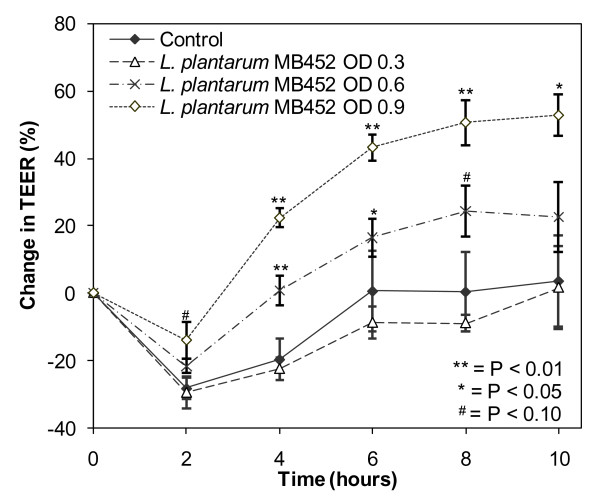
**Change in trans-epithelial electrical resistance (TEER) across confluent Caco-2 monolayers (5 days old) over time in the presence of different optical densities of *L. plantarum *MB452**. The change in TEER is the percentage change compared to the initial TEER for each monolayer. The values plotted are the means for four monolayers and the error bars show the SEM. OD = the starting optical density of the *L. plantarum *cultures at 600 nm.

The ability of *L. plantarum *MB452 to increase intestinal barrier function was investigated by determining the effect on TEER using different concentrations of *L. plantarum *MB452 (Figure 1). At an OD_600 nm _of 0.3 (7 × 10^7 ^CFU/mL) *L. plantarum *MB452 did not cause an increase in TEER compared to the untreated controls. At an OD_600 nm _of 0.6 (1.8 × 10^8 ^CFU/mL) *L. plantarum *MB452 caused an increase in TEER of 15-20% compared to the untreated controls at 4 and 6 hours. At an OD_600 nm _of 0.9 (3 × 10^8 ^CFU/mL) *L. plantarum *MB452 caused an increase in TEER of 42-51% compared to the untreated controls from 4 to 10 hours. The effect of *L. plantarum *MB452 on TEER was 19-27% higher at an OD_600 nm _of 0.9 compared to OD_600 nm_of 0.6 (P < 0.05 from 4 to 8 hours). Similarly, the effect of *L. plantarum *MB452 on TEER was 23-33% higher at an OD_600 nm _of 0.6 compared to OD_600 nm _of 0.3 (P < 0.05 from 4 to 8 hours).

The drop in TEER for all treatments between 0 and 2 hours observed in all assays was likely due to the Caco-2 cell monolayers being disturbed by the change in media during the sample addition after the initial readings. The increase in TEER over time for the control Caco-2 cells was likely due to an increase in nutrient availability after the fresh media was added at the beginning of the experiment. The increases in TEER caused by *L. plantarum *MB452 were additional to those observed with fresh media.

**Figure 2:**
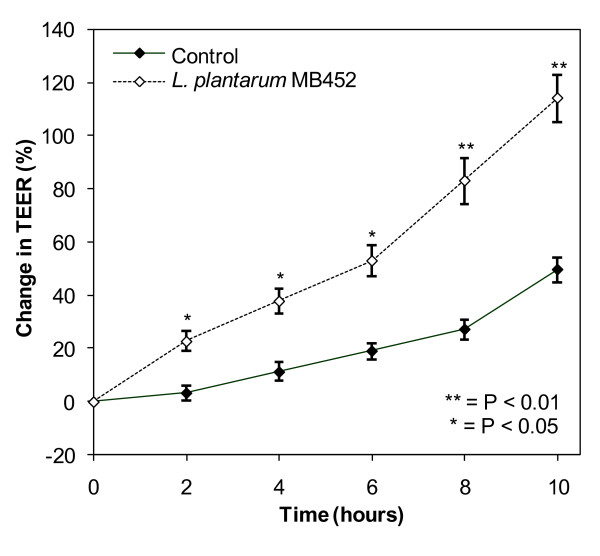
**Change in trans-epithelial electrical resistance (TEER) across differentiated Caco-2 monolayers (18 days old) in the presence of *L. plantarum *MB452 (OD_600 nm _0.9)**. The change in TEER is the percentage change compared to the initial TEER for each monolayer. The values plotted are the means for four monolayers and the error bars show the SEM.

*L. plantarum *MB452 was also able to increase the TEER by 20 at 2 hours to 64% at 10 hours across differentiated Caco-2 cells (18 days old; Figure 2) in the same manner as for confluent, undifferentiated cells (5 days old; Figure 1). A differentiated, polarised Caco-2 cell monolayer better represents the human intestinal barrier than confluent undifferentiated Caco-2 cells. The tight junctions between the differentiated Caco-2s were better formed than the undifferentiated Caco-2s (higher initial TEER readings), were less affected by the media addition (no initial drop in TEER) and had less variation between replicates (lower SEM values).

### *L. plantarum *MB452 altered the expression of genes involved tight junction formation

The ability of *L. plantarum *MB452 to alter gene expression in intestinal epithelial cells was measured using global gene expression analysis. The analysis indicated that 1,181 Caco-2 cell genes were differentially expressed (fold change > 1.2, modified-P < 0.05) when co-cultured with *L. plantarum *MB452; the expression levels of 554 genes were increased and 627 genes were decreased. The relatively low fold-change cut-off of 1.2 was selected because the comparison was between untreated healthy cells and healthy cells treated with potential beneficial bacteria so large differences (as seen when comparing healthy cells to a disease model) were not expected. The ten Ingenuity Pathway Analysis (IPA) functional groups with the most differentially expressed genes and the Gene Ontology categories with the lowest P-values are summarised in Additional File 1 Tables S1 and S2.

**Figure 3:**
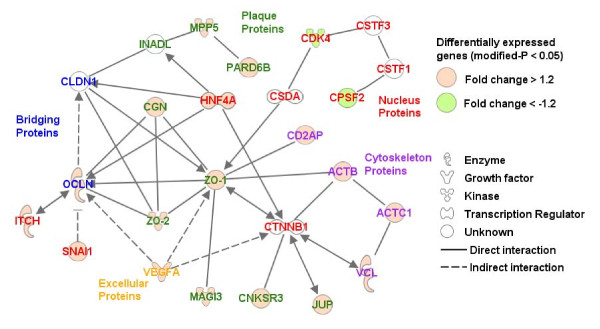
**Network of genes involved in tight junction formation that were differentially expressed by Caco-2 cells after being co-cultured with *L. plantarum *MB452 (OD_600 nm _0.9) for 10 hours**. Genes are represented as nodes and the biological relationship between two nodes is represented as an edge. All edges are supported by at least one reference from the literature. Red and green colored nodes indicate genes that have increased or decreased expression, respectively, in response to *L. plantarum *MB452. The colors of the gene names indicate the role the encoded proteins in relation to tight junctions.

Of the genes that were differentially expressed in response to *L. plantarum *MB452, 19 were involved in tight junction formation (Table 1). Analysis of KEGG pathways using EASE showed that the tight junction pathway was one of four pathways that was enriched with differentially expressed genes (P and global FDR < 0.05; Additional File 1 Table S3). The molecular interactions between these genes were visualised in an IPA network diagram (Figure 3). The nodes with the most interactions are those that represent the genes for occludin, ZO-1, ZO-2 and cingulin.

**Table 1:** Caco-2 cell genes involved in intracellular junction complex formation that were differentially expressed in the microarray analysis after co-culturing with L. plantarum MB452 (OD600 nm 0.9) for 10 hours.

Gene Name	Symbol	Refseq ID	Fold Change	Moderated	Description of role in relation to tight junctions
occludin	OCLN	NM_002538	1.39	0.004	tight junction bridging protein
vascular endothelial growth factor A	VEGFA	NM_001025366	1.39	0.002	cytokine that indirectly regulates tight junction formation and strengthening
actin beta	ACTB	NM_001101	1.33	0.005	structural constituent of cytoskeleton
cingulin	CGN	NM_020770	1.29	0.024	tight junction plaque protein associated with occludin
par-6 partitioning defective 6 homolog beta	PARD6B	NM_032521	1.27	0.009	tight junction plaque protein associated with claudins and involved in cell polarization
actin alpha cardiac muscle 1	ACTC1	NM_005159	1.25	0.015	structural constituent of cytoskeleton
itchy homolog E3 ubiquitin protein ligase	ITCH	NM_031483	1.25	0.011	ubiquitin-ligase molecule that regulates occludin degradation
junction plakoglobin	JUP	NM_002230	1.24	0.010	major cytoplasmic protein that forms a complex with cadherins
CNKSR family member 3	CNKSR3	NM_173515	1.24	0.006	tight junction plaque protein associated with JAMs
snail homolog 1	SNAI1	NM_005985	1.24	0.033	intracellular component that indirectly inhibits occuldin production
hepatocyte nuclear factor 4 alpha	HNF4A	NM_178849	1.24	0.021	transcription regulator that acts on occuldin
zona occludens 1 (tight junction protein 1)	ZO-1	NM_003257	1.23	0.013	tight junction plaque protein associated with occludin, JAMs and claudins
zona occludens 2 (tight junction protein 2)	ZO-2	NM_004817	1.23	0.054	tight junction plaque protein associated with occludin and claudins that acts as a guanylate kinase and also found in the nucleus
CD2-associated protein	CD2AP	NM_012120	1.22	0.012	scaffolding molecule that regulates the actin cytoskeleton
vinculin	VCL	NM_003373	1.22	0.027	cytoskeletal protein
membrane associated guanylate kinase 3	MAGI-3	NM_152900	1.21	0.044	tight junction plaque protein a and guanylate kinase
membrane protein, palmitoylated 5	MPP5	NM_022474	1.20	0.014	tight junction plaque protein associated with claudins and guanylate kinase involved in tight junction organization
cleavage and polyadenylation specific factor 2	CPSF2	NM_017437	-1.22	0.022	transcription regulator that decreases tight junction stability
cyclin-dependent kinase 4	CDK4	NM_000075	-1.30	0.011	transcription regulator that decreases tight junction stability

The expression levels of seven genes was also quantified using real-time PCR (qRT-PCR) and was compared with the gene expression data obtained using microarray analysis (Table 2). Of the 5 genes that had increased expression in the microarray analysis, occludin and cingulin were shown to have increased expression in response to *L. plantarum *MB452 using qRT-PCR. Three other genes were differentially expressed in the microarray analysis but not in the qRT-PCR analysis. The CLDN3 gene was not differentially expressed in the microarray or qRT-PCR analyses. The GJA7 gene had decreased expression in the microarray analysis (fold change -1.39) and increased expression in the qRT-PCR analysis (fold change 3.08). The variation between the gene expression results obtained between the two techniques is likely due to the fact that the qRT-PCR probes used did not recognise the same transcripts as the microarray probes, which is the most common reason for discrepancies between results of the two methods. It has been shown that when qRT-PCR and microarray probes recognise the same transcripts there is an accordance of results with 87% of genes; whereas, when the qRT-PCR and microarray probes do not recognise the same transcripts there is an accordance of only 41% [24]. These data indicated an accordance for 43% of the genes (3/7 genes) using the two methods.

**Table 2:** Comparison between microarray and qRT-PCR analysis of Caco-2 cells genes after co-culturing with L. plantarum MB452 (OD600 nm 0.9) for 10 hours.

Gene	Microarray fold change	qRT-PCR fold change
OCLN	1.39^1^	2.59^2^
ACTB	1.33^1^	1.06
CGN	1.29^1^	3.23^2^
ZO-1	1.23^1^	1.17
ZO-2	1.23^1^	1.46
CLDN3	1.01	1.23
GJA7	-1.39^1^	3.08^2^

^1 ^Modified P-value < 0.05

^2 ^P-value < 0.05

### *L. plantarum *MB452 altered the expression of other tight junction associated genes

Eight genes encoding for cytoskeleton tubulin proteins had decreased expression levels (fold change -1.20 to -1.45) in Caco-2 cells treated with *L. plantarum *MB452 (Table 3). Similarly, seven genes encoding for protein degrading proteasomes had decreased expression levels (fold change -1.21 to -1.28) in Caco-2 cells treated with *L. plantarum *MB452 (Table 3).

**Table 3:** Caco-2 cell tubulin and proteasome genes that were differentially expressed (modified-P < 0.05) in the microarray analysis after co-culturing with L. plantarum MB452 (OD600 nm 0.9) for 10 hours.

Gene Name	Symbol	Refseq ID	Fold Change
tubulin, alpha 1b	TUBA1B	NM_006082	-1.45
tubulin, alpha 1c	TUBA1C	NM_032704	-1.35
tubulin, alpha 3d	TUBA3D	NM_080386	-1.22
tubulin, alpha 4a	TUBA4A	NM_006000	-1.27
tubulin, beta	TUBB	NM_178014	-1.20
tubulin, beta 3	TUBB3	NM_006086	-1.20
tubulin, beta 6	TUBB6	NM_032525	-1.30
tubulin, beta 2c	TUBB2C	NM_006088	-1.35
proteasome, alpha subunit, 5	PSMA4	NM_002789	-1.24
proteasome, beta subunit, 1	PSMB1	NM_002793	-1.21
proteasome, beta subunit, 6	PSMB6	NM_002798	-1.22
proteasome, beta subunit, 7	PSMB7	NM_002799	-1.28
proteasome, 26 s subunit, 5	PSMC5	NM_002805	-1.24
proteasome, 26 s subunit non-ATPase, 12	PSMD12	NM_002816	-1.25
proteasome, activator subunit, 2	PSME2	NM_002818	-1.24

### *L. plantarum *MB452 visually increased the abundance of tight junction proteins

**Figure 4:**
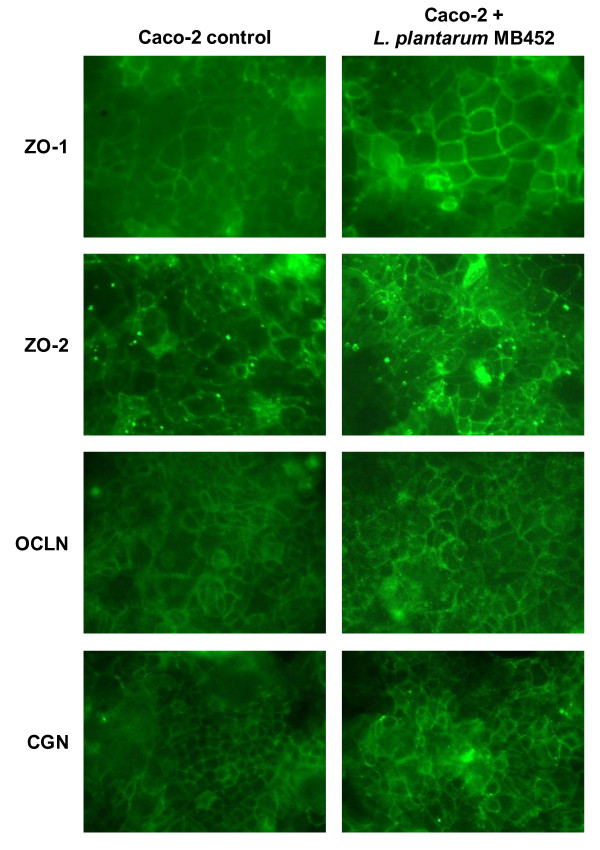
**Fluorescent microscopy images of immuno-stained tight junction proteins of confluent Caco-2 cells (6 days old) untreated or treated with *L. plantarum *MB452 (OD_600 nm _0.9) for 8 hours**. Treatments were carried out in quadruplicate and the images shown are typical. ZO-1: zonula occluden 1; ZO-2 zonula occluden 2; OCLN: occludin; CGN: cingulin.

Using fluorescent microscopy the intensity of the immuno-stained ZO-1, ZO-2 occludin and cingulin proteins appeared higher in the Caco-2 cells treated with *L. plantarum *MB452 than in the untreated controls (Figure 4). This indicated that the changes in gene expression observed were supported by changes in tight junction-associated protein intensity.

## Discussion

As hypothesised, this study showed that *L. plantarum *MB452 altered the expression levels of tight junction-related genes in healthy intestinal epithelial cells. Of the tight junction bridging proteins, occludin mRNA abundance was higher in the presence of *L. plantarum *MB452. The over-expression of the occludin protein has been linked to increased TEER [25], and based on the findings of this study, increased occludin gene expression may contribute to the ability of *L. plantarum *MB452 to enhance tight junction integrity. In support of this, genes encoding for the occludin-associated plaque proteins, ZO-1 and ZO-2 and cingulin, also had increased expression levels in the presence of *L. plantarum *MB452. The zonula occludens bind to the cytoplasmic end of occludin and form the scaffolding to link occludin to the actin cytoskeleton [26]. The increased expression levels of the occludin, ZO-1, ZO-2 and cingulin genes appeared to correlate with increased intensity of their proteins as indicated by the fluorescent microscopy images.

In contrast, other genes that had increased transcript levels in the presence of *L. plantarum *MB452 are known to be involved in tight junction disassembly. The gene encoding ITCH, an ubiquitin-ligase molecule, had increased expression levels in the presence of *L. plantarum *MB452; however, the ITCH protein is known to contribute to the degradation of occludin [27]. The increased expression of the ITCH gene may lead to an increase in the turnover of occludin protein and, therefore, may have contributed to the increased occludin mRNA noted in this data. The gene encoding the SNAI1 protein also had increased expression in the presence of *L. plantarum *MB452; however, the SNAI1 protein is known to bind to occludin and claudin genes promoters suppressing their expression [28]. Although these two genes, ITCH and SNAI1, have been linked to tight junction disassembly, 17 out of the 19 tight junction-related genes with increased expression levels in response to *L. plantarum *MB452 exposure contribute to tight junction stability; therefore, the cumulative effect would most likely be enhanced intestinal barrier function.

The 'tightness' of tight junctions is commonly thought to be, at least partly, due to claudins, which are a set of bridging proteins; however, none of the claudin genes were differentially expressed in response to *L. plantarum *MB452. Decreases in the abundance of claudin-2, -3 and -4 proteins (measured using western blotting) have been associated with a decrease in TEER [29]. Another study showed that a decrease in TEER was associated with altered cellular localisation of claudin-1 and -5, but not altered abundance [30], so it is possible that *L. plantarum *MB452 may have altered the distribution of claudin proteins without changing gene expression and/or protein abundance.

The results of this study showed that *L. plantarum *MB452 enhanced the expression of 19 genes involved in the tight junction signalling pathway in healthy cells. A previous study showed that *L. plantarum *CGMCC 1258 is able to protect against the disruption of four tight junction proteins caused by Enteroinvasive *E. coli *ATCC 43893 (serotype O124:NM) [17]. However, another study looking at the effect of *L. plantarum *ATCC202195 on the expression of genes in Caco-2 cells challenged with Enteroinvasive *E. coli *ATCC43893 (serotype O124:NM) did not report any changes in tight junction gene expression [31]. This suggests that the *L. plantarum *protection against tight junction disruption was not due to it altering host gene expression, and was likely due to it inhibiting the action of the pathogen in that study.

The ability to enhance the expression of tight junction-related genes is not common to all *L. plantarum *strains. In addition to the study that showed that *L. plantarum *ATCC202195 nullifies changes in Caco-2 cell gene expression induced by Enteroinvasive *E. coli *ATCC43893 (serotype O124:NM) [31], other published studies investigating whole genome expression in response to *L. plantarum *have shown that, *L. plantarum *WCFSI induces increased expression of genes involved in lipid metabolism and cellular growth and development in healthy human duodenum [32] and *L. plantarum *(strain not given) alters the NF-κB pathway to limit inflammatory responses in healthy human duodenum [33]. However, in these published studies only a few tight junction-related genes had altered expression levels when exposed to *L. plantarum*, for example increased expression of the ZO-2 gene, so they are unlikely to contribute to changes in tight junction integrity, compared to the changes in 19 tight junction genes induced by *L. plantarum *MB452 reported in this study. This is not surprising since strains of *L. plantarum *can have differing effects on intestinal barrier function *in vitro*, from neutral (cause no increase in TEER) to beneficial (cause substantial increase in TEER; unpublished results), and thus, it is likely that different strains may also have different effects on epithelial cell gene expression.

The observed increase in intestinal barrier function induced by *L. plantarum *MB452 may also be, at least partly, due to changes in intestinal epithelial cell gene expression that have an indirect effect on tight junction stability. Eight genes encoding for tubulins had lower expression levels in response to *L. plantarum *MB452. A high turnover in tubulin synthesis has been linked to the disassembly of tight junctions [34]; thus, the reduced expression levels of these genes may account for the positive effect of *L. plantarum *MB452 on intestinal barrier function. Similarly, seven genes encoding for proteasome subunits had lower mRNA abundance in the presence of *L. plantarum *MB452. Proteasomes, which are large protein complexes responsible for breaking down surplus or damaged proteins, have previously been linked to tight junction degradation, and proteasome inhibitors can prevent degradation of occludin [35] and ZO-2 [36]. The reduction in proteasome gene expression induced by *L. plantarum *MB452 may be an additional mechanism by which tight junction integrity is enhanced.

Several of the tight junction-related genes with altered expression induced by *L. plantarum *MB452 may also be involved in reducing cell proliferation. For example, ZO-1, which had increased gene expression in the presence of *L. plantarum *MB452, is a 'dual location protein' involved in the regulation of cell proliferation. The ZO-1 protein binds to the CSDA protein (also known as ZONAB) and sequesters it to tight junctions, and removal of the CSDA protein from nucleus in this way results in a reduction in the CDK4 protein [37]. Therefore, an increase in ZO-1 gene expression may lead to a decrease in CDK4 gene expression as seen here (Figure 3), which highlights the link between the formation of tight junctions and a reduction in cell proliferation [37]. Additionally, *L. plantarum *MB452 reduced the expression of the CPSF2 gene, which encodes a protein which is part of the CSTF-CPSF-SYMPK complex, that regulates cell-cycle related gene expression and promotes cell proliferation [38]. Together with the decreased expression of tubulin genes, these effects of *L. plantarum *MB452 on the ZO-1, CDK4 and CPSF2 genes may lead to decreased cell proliferation and contribute to the reported anti-proliferative effect of the VSL#3 product [39].

*L. plantarum *MB452 did not alter the expression levels of other genes and pathways that have been affected by some probiotic bacteria, such as the NF-κB pathway [33], PPARγ [40,41], innate immune response pathway [42], or human β defensin-2 [43]. This indicates that, unlike some other probiotic bacteria, *L. plantarum *MB452 does not seem to exert its beneficial effect by regulating host immune responses in healthy intestinal cells.

In this study using *L. plantarum *MB452 alone, only certain effects previously associated with VSL#3 were observed. VSL#3 is a mixture of *L. plantarum, L. casei, L. acidophilus, L. delbrueckii *subspecies *bulgaricus, B. longum, B. breve, B. infantis *and *Streptococcus thermophilus*, and is likely that each bacterial species has a range of effects. A previous study indicated that of the bacterial strains present in VSL#3, the culture supernatant of *B. infantis *was associated with the greatest increase in TEER across Caco-2 cells compared to untreated controls [15]. Of the VSL#3 lactobacilli, *L. plantarum *MB452 produced the supernatant with the greatest effect of TEER, which is in agreement with previous work that showed the beneficial effects of *L. plantarum *MB452 supernatant [44]. Other studies indicated that the anti-inflammatory effects of VSL#3 are, at least partially, due to VSL#3 bifidobacteria decreasing the abundance of the pro-inflammatory cytokine IL-8 [45] and *L. casei *in VSL#3 reducing the abundance the pro-inflammatory cytokine interferon gamma-induced protein 10 [46]. The genes encoding for these cytokines were not altered in response to *L. plantarum *MB452 in the present study.

## Conclusions

The data presented in this study shows that a probiotic, *L. plantarum *MB452, enhanced intestinal barrier function by affecting the expression of genes in the tight junction signalling pathway in health intestinal epithelial cells, in particular the genes encoding occludin and its associated plaque proteins, ZO-1, ZO-2 and cingulin. Further studies will investigate the function of these key genes and evaluate their role in *L. plantarum *MB452 mediated changes in intestinal barrier function. These results also highlight that changes in intestinal barrier function may also be linked to changes in tubulin and/or proteasome gene expression. Further targeted studies will investigate whether these gene expression changes are important in the observed enhanced intestinal barrier function, and, if so, the mechanisms involved. The long-term aim is to understand the way in which bacteria increase tight junction integrity in order to develop probiotic foods that enhance intestinal barrier function and therefore improve human wellness.

## Methods

### Bacterial strains

*Lactobacillus plantarum *MB452 [47] was isolated from VSL#3 (Orphan Australia Pty Ltd, Berwick, Australia). A sachet of VSL#3 powder was suspended in 50 mL of sterile water and the culture was streaked on MRS agar plates (de Man, Rogosa and Sharpe Broth, Difco, Sparks, USA) and incubated aerobically at 30°C, 37°C and 42°C and anaerobically at 37°C. Colony morphologies were recorded and sample colonies from each plate were sub-cultured into Brain Heart Infusion broth (Difco, USA) with 30% glycerol for storage at -85°C. As described in Additional File 2, colonies with similar morphology were compared using pulse-field gel electrophoresis and representatives of each profile were identified based on their 16 s rRNA sequences.

### Mammalian cell culture

Caco-2 cell (HTB-37; ATCC) stock cultures were grown in T75 flasks in M199 tissue culture medium with 10% foetal bovine serum (GIBCO, Invitrogen Corporation, Auckland, NZ), 1% non-essential amino acids (MEM non-essential amino acids 100× solution, Sigma-Aldrich, St Louis, USA) and 1% penicillin-streptomycin (10,000 units penicillin G sodium salt and 10000 g streptomycin sulphate in 0.85% saline, GIBCO, Invitrogen Corporation, Auckland, NZ) at 37 C in 5% CO_2_. The media was replaced every 3 to 4 days and the cells were subcultured weekly at a ratio of 1:3. Caco-2 cells with a passage number of 30 to 35 were used for all experiments.

### Trans-epithelial electrical resistance assay

Caco-2 cells were seeded onto 14 mm collagen membrane inserts (Cellagen™ Discs CD-24, MP Biomedicals, Ohio, USA) at a density of 10^5 ^cells/insert. Each insert was placed in a well in a 12-well plate with 1 mL of media in the bottom and 250 µL media in the top. The media was replaced every 2 to 3 days. Confluent Caco-2 monolayers (5 days old) were used for the majority of the TEER experiments; except for the TEER experiment done in parallel with the gene expression experiment where differentiated Caco-2 monolayers were used (18 days old). All Caco-2 monolayers had initial TEER values of greater than 300 ohms.cm^2^. The Caco-2 monolayers were prepared the day before the TEER assay by removing the media, washing three times with PBS and adding M199 with 1% non-essential amino acids (without foetal bovine serum and penicillin-streptomycin) to ensure growth of the bacterial cells. To prepare the bacteria for the TEER assay, an overnight culture of bacterial cells (MRS broth, 37°C, 5% CO_2_) were collected by centrifugation (12,000 rpm for 5 minutes in a micro centrifuge), washed in phosphate buffered saline (PBS, pH 7.2), and suspended in M199 with 1% non-essential amino acids to the required optical density at 600 nm. After the initial resistance readings were taken on the day of the experiment, the media was removed from the top of the Caco-2 monolayers and replaced with the treatment solutions. For the controls, the spent media was replaced with fresh media (M199 and 1% non-essential amino acids). Each treatment was performed in quadruplicate and each assay was repeated three times.

Every two hours, each insert was lifted into an electrode chamber (ENDOHM-12 tissue culture chamber, World Precision Instruments, Florida, USA) using sterile tweezers and the resistance was measured using a voltohmmeter (EVOM Epithelial Tissue Voltohmmeter, World Precision Instruments, Florida, USA). The TEER was calculated from the resistance using the formula: TEER (Ω cm^2^) = (resistance (Ω) – background resistance (Ω)) × membrane area (cm^2^), where the background resistance was 14 and the membrane area was 1.54 cm^2^. The change in TEER for each insert was calculated using the following formula: change in TEER (%) = TEER (Ω cm^2^)/initial TEER (Ω.cm^2^) - 100 (%). The mean change in TEER was plotted against time, with the error bars showing the SEM. Treatments were compared in GenStat (Version 11.1.0.1575) using residual maximum likelihood analysis with an unstructured covariance model to take account of the repeated measures. Statistical differences between treatments were declared at a probability less than 0.05 whilst a probability between 0.05 and 0.1 was considered to represent a trend.

### Gene expression analysis

Caco-2 cells were seeded into all wells in 6-well plates at a density of 3 × 10^5 ^cells/well. The media was replaced every 3-4 days and the Caco-2 monolayers were grown for 18 days to allow them to differentiate. Six wells were treated with *L. plantarum *MB452 (OD 600 nm of 0.9) suspended in cell culture media (M199 and 1% non-essential amino acids) and six wells were treated with control media. After 10 hours of exposure (37°C, 5% CO_2_) the treatment solutions were removed and the monolayers were rinsed with PBS.

The total RNA was extracted from the Caco-2 cells using TRIzol, (Invitrogen, Auckland, New Zealand) and purified using RNeasy mini columns (QIAGEN, San Diego, CA, USA). An equal amount of RNA from three wells of the same treatment was pooled together to yield enough RNA for the gene expression analysis (microarray and qRT-PCR); two control pools and two pools treated with *L. plantarum *MB452. Equal amounts of RNA from all 12 wells were pooled together to make the reference RNA sample. A similar experimental design previously gave biologically relevant results [48,49]. RNA samples were labelled, amplified and hybridised to Agilent Technologies 44 k whole human genome oligonucleotide arrays (G4112A) according to the manufacturer's instructions. The Limma package in Bioconductor was used to analyse the microarray data [50]. Genes with a fold change greater than 1.2 and a modified p-value less than 0.05 were considered differentially expressed. Differentially expressed genes were clustered into functional groups and pathways using Ingenuity Pathway Analysis (IPA version 7.1; Ingenuity Systems Inc., Redwood City, CA, USA), and Gene Ontology categories and KEGG pathways using EASE (version 2.0)[51]. The raw microarray data have been deposited in the NCBI Gene Expression Omnibus under the GEO accession number GSE18372. Full details of the methods are given in Additional File 3.

The expression of tight junction-related genes differentially expressed from the microarray analysis was confirmed using qRT-PCR. The expression of seven target genes relative to three reference genes was assessed using the standard curve method. The reference genes (GAPD, SDHA and YWHAZ) were chosen based on the findings of Vandesompele *et al *[52] and their log ratios in the microarray data (close to 1; not differentially expressed). Five target genes (ZO-1, ZO-2, OCLN, CGN and ACTB) were chosen from the tight junction-related genes that were differentially expressed (all up-regulated) in the microarray analysis. The two other target genes, GJA7 and CLDN3, were chosen to be included because they were down-regulated and not differentially expressed, respectively, in the microarray analysis. The analysis was carried out as described in Additional File 3 and the data was analysed using Relative Expression Software Tool 2008 (version 2.0.7) with efficiency correction [53].

### Fluorescent microscopy

Caco-2 cells were grown on Lab Tek II Chamber Slides with Permanox™ coating (Nalge Nunc International Corp, Naperville, IL, USA) for 6 days until confluent. Caco-2 cells were treated with *L. plantarum *MB452 (OD 600 nm 0.9) or control media for 8 hours (n = 4 per treatment per antibody). After treatment, Caco-2 cells were rinsed twice with PBS, fixed in either 4% (w/v) paraformaldehyde for 20 minutes (for CGN and ZO-1) or ice cold 70% ethanol (for ZO-2 and OCLN), quenched with 50 mM NH_4_Cl (in PBS) for 15 minutes, and blocked with blocking solution (2% (v/v) foetal bovine serum, 1% sheep serum albumin, 0.1% Triton X-100, 0.05% Tween 20 in PBS, pH 7.2) for 20 minutes. Caco-2 cells were then immuno-stained with the primary antibodies (2.5 µg/mL rabbit anti-ZO-1, 1.25 µg/mL rabbit anti-ZO-2, 2.5 µg/mL rabbit anti-occludin, 1 µg/mL rabbit anti-cingulin; Zymed, Invitrogen, NZ) in blocking solution for 1 hour, followed by a PBS wash (0.1% Triton X-100, 0.05% Tween 20 in PBS) to reduce non-specific staining, and the secondary antibody, Alexa Fluor 488 goat anti-rabbit IgG (5 µg/mL for ZO-2, 10 µg/mL for rest; Invitrogen, NZ) in blocking solution for 1 hour. The slides were imaged with a fluorescent microscope (Leica DM2500 microscope, Leica DFC420C camera) with the following settings: exposure 1.1 ms, saturation 2.25, gamma 1.52, gain 8.4× and magnification 40×. The images were viewed using LAS Image Overlay software (Leica Application Suite v1.8.2).

## Authors' contributions

RCA, ALC, WCM and NCR designed the research; RCA, ALC, ZP, MJM and WJK conducted some of the research; All authors analysed the data; RCA and NCR wrote the paper; RCA had primary responsibility for final content; All authors read and approved the final manuscript.

## Supplementary Material

Additional file 1 **Summary of microarray analysis**. Contains Tables S1, S2 and S3 which summarise the differentially expressed IPA Functional Groups, Gene Ontology categories and KEGG pathways, respectively. Click here for file

Additional file 2 **Identification of *L. plantarum *MB 452 from VSL#3**. Describes the Pulse-field gel electrophoresis and 16 s sequencing methods used to identify *L. plantarum *MB 452. Click here for file

Additional file 3 **Analysis of gene expression of Caco-2 cells treated with *L. plantarum *MB452**. Describes the microarray analysis and qRT-PCR analysis. Include Table S4 showing the qRT-PCR primers. Click here for file
