# Supplementary material for: Lactobacillus plantarum MB452 enhances the function of the intestinal barrier by increasing the expression levels of genes involved in tight junction formation
Source: BMC Microbiol. 2010 Dec 9;10:316. doi: 10.1186/1471-2180-10-316 (PMC3004893; doi:10.1186/1471-2180-10-316)
Supplement: Additional file 1 — Summary of microarray analysis. Contains Tables S1, S2 and S3 which summarise the differentially expressed IPA Functional Groups, Gene Ontology categories and KEGG pathways, respectively. [file 1471-2180-10-316-S1.DOC]

**Additional File 1 – Summary of Microarray Data Analysis**

**Table S1.** Top ten Functional Groups (Ingenuity Pathway Analysis) differentially expressed in Caco-2 cells in response to co-culturing with *L. plantarum* MB 452 for 10 hours.

| **Functional Group** | **p-value range** | **Number of differentially expressed genes** |
| --- | --- | --- |
| Cancer | 1.9 x 10^-16^ – 1.4 x 10^-03^ | 206 |
| Cell Signalling | 8.1 x 10^-22^ – 1.3 x 10^-03^ | 204 |
| Cellular Growth and Proliferation | 2.4 x 10^-20^ – 1.4 x 10^-03^ | 187 |
| Cell Death | 6.6 x 10^-23^ – 1.4 x 10^-03^ | 173 |
| Gene Expression | 2.4 x 10^-15^ – 9.1 x 10^-04^ | 138 |
| Cellular Development | 7.8 x 10^-10^ – 1.0 x 10^-04^ | 128 |
| Cell Cycle | 4.4 x 10^-20^ – 1.4 x 10^-03^ | 106 |
| Reproductive System Disease | 1.4 x 10^-08^ – 1.4 x 10^-03^ | 98 |
| Cellular Movement | 2.3 x 10^-06^ – 1.4 x 10^-03^ | 89 |
| Cell Morphology | 3.2 x 10^-12^ – 1.3 x 10^-03^ | 82 |

**Table S2.** Top ten Gene Ontology categories differentially expressed in Caco-2 cells in response to co-culturing with *L. plantarum* MB 452 for 10 hours.

| **Gene Category** | **P-value** |
| --- | --- |
| GO:0005622 intracellular | 2.39 x 10^-11^ |
| GO:0044424 intracellular part | 2.47 x 10^-9^ |
| GO:0005634 nucleus | 5.70 x 10^-8^ |
| GO:0043170 macromolecule metabolic process | 6.95 x 10^-8^ |
| GO:0043231 intracellular membrane-bound organelle | 9.14 x 10^-8^ |
| GO:0043227 membrane-bound organelle | 9.95 x 10^-8^ |
| GO:0043226 organelle | 2.53 x 10^-7^ |
| GO:0043229 intracellular organelle | 3.77 x 10^-7^ |
| GO:0043283 biopolymer metabolic process | 8.82 x 10^-7^ |
| GO:0008134 transcription factor binding | 4.13 x 10^-6^ |
| GO:0005515 protein binding | 8.40 x 10^-6^ |

**Table S3.** KEGG pathways differentially expressed in Caco-2 cells in response to co-culturing with *L. plantarum* MB 452 for 10 hours.

| **KEGG pathway** | **P-value** | **Official Gene Symbol** |
| --- | --- | --- |
| hsa05220:Chronic myeloid leukemia | 0.0093 | CBL; CCND1; CDK6; CTBP1; CTBP2; E2F2; E2F3; GAB2; NFKBIA; TGFBR2 |
| hsa04115:p53 signaling pathway | 0.0354 | BAX; BBC3; CCNB1; CCND1; CDK6; IGFBP3; RRM2; TNFRSF10B |
| hsa04530:Tight junction | 0.0405 | ACTN4; CGN; CLDN3; CSDA; MYH9; OCLN; PPP2CA; PRKCZ; TJP1; TJP2; VAPA; YES1 |
| hsa04920:Adipocytokine signaling pathway | 0.0484 | IRS1; NFKBIA; NFKBIE; PCK1; POMC; PPARA; PRKAB2; TNFRSF1A |
