# Supplementary material for: Lactobacillus plantarum MB452 enhances the function of the intestinal barrier by increasing the expression levels of genes involved in tight junction formation
Source: BMC Microbiol. 2010 Dec 9;10:316. doi: 10.1186/1471-2180-10-316 (PMC3004893; doi:10.1186/1471-2180-10-316)
Supplement: Additional file 2 — Identification of L. plantarum MB 452 from VSL#3. Describes the Pulse-field gel electrophoresis and 16 s sequencing methods used to identify L. plantarum MB 452. [file 1471-2180-10-316-S2.DOC]

**Additional File 2.** Identification of *L. plantarum* MB 452 from VSL#3

**Pulse-field gel electrophoresis**

Colonies with similar morphology were compared using pulse-field gel electrophoresis to determine if they were the same organism. A 1% (v/v) inoculum of a stationary phase culture was added to 10 mL of fresh MRS broth and allowed to grow overnight. Cells from 1.5 ml samples of the culture were harvested by centrifugation, washed once in 1M NaCl, 10 mM Tris.Cl (pH 7.6), and suspended in 300 µL of the same solution. This was mixed with an equal volume of 2% low melting point agarose (Bio-Rad Laboratories, Richmond, CA.) in 0.125 M EDTA (pH 7.6), and left to solidify in moulds (Bio-Rad). Agarose blocks were removed from the moulds and the cells were lysed by incubating the agarose blocks for 18 hours at 37°C in EC buffer (1 M NaCl, 6 mM Tris.Cl, 100 mM EDTA, 1% (w/v) sarkosyl, pH 7.6) containing 5 mg/mL lysozyme. The agarose blocks were then incubated with proteinase K (1 mg/mL in 0.5 M EDTA, 1% sarkosyl, pH 8.0) for 24 hours at 37°C, and with 1 mM phenylmethylsulfonyl fluoride (PMSF) in TE 10/1 (10 mM Tris.Cl, 1 mM EDTA, pH 8.0) for 2 hours at 37°C. After treatment the agarose blocks were stored in TE 10/100 (10 mM Tris.Cl, 100 mM EDTA, pH 8.0) at 4°C until required.

Slices (1-2 mm) were cut from the agarose blocks with a sterile coverslip, and washed twice with gentle shaking in TE 10/0.1 (10 mM Tris.Cl, 0.1 mM EDTA, pH 8.0) at room temperature. Slices were transferred to microcentrifuge tubes, washed once at 4°C with restriction enzyme buffer, and incubated for 16-18 hours with the appropriate restriction enzyme in a total volume of 100 µL. The enzymes used to differentiate strains of the lactobacilli were AscI and I-CeuI (New England Biolabs, Beverly, MA). Following digestion, the slices were washed once with TE 10/1 for one hour at 4°C and loaded into the wells of a 1% agarose gel (Bio-Rad pulsed field certified agarose prepared in 0.5X Tris-borate buffer). The wells were sealed with agarose and the gel run in 0.5X Tris-borate buffer using a CHEF DR III pulsed-field gel electrophoresis apparatus and model 1000 mini chiller (Bio-Rad). Gels were run at 200V for 20 hours at 14°C, and with the pulse time ramped from 1 to 30 seconds for AscI digests, and from 10 to 100 seconds for I-CeuI digests. Multimers of bacteriophage lambda prepared for use as pulsed-field gel molecular size standards (New England Biolabs) were used to give an indication of fragment sizes. Gels were stained with Gelstar nucleic acid gel stain (Cambrex Bio Science, Rockland, ME), destained with water, and photographed using a Kodak Gel Logic 200 Imaging System (Eastman Kodak, Rochester, NY).

**16s sequencing**

Representatives of each pulse-field gel electrophoresis profile were identified based on their 16s rRNA sequences. From the isolated genomic DNA the 16S rRNA was amplified using with FD1 (5'- AGAGTTTGATCCTGGCTCAG-3') and RD1 (5’-AAGGAGGTGATCCAGCC-3’) primers and PCR supermix (Invitrogen, Auckland) using a Thermo Hybaid PX2 thermocycler (Thermo Electron Corporation) and purified using a QIAquick PCR purification kit (QIAGEN). The florescent labelled DNA was prepared using PCR with Big Dye Terminator v3.1 Cycle Sequencing kit (Applied Biosystems) and purified using ethanol/EDTA precipitation. The DNA sequencing was carried out by the Allan Wilson Centre Genome Service at Massey University, Palmerston North, New Zealand. The DNA sequence contigs were aligned using the ContigExpress Vector NTI software (Invitrogen). The determined sequences were compared to known bacterial sequences using the NCBI Blast database.
