# Supplementary material for: Lactobacillus plantarum MB452 enhances the function of the intestinal barrier by increasing the expression levels of genes involved in tight junction formation
Source: BMC Microbiol. 2010 Dec 9;10:316. doi: 10.1186/1471-2180-10-316 (PMC3004893; doi:10.1186/1471-2180-10-316)
Supplement: Additional file 3 — Analysis of gene expression of Caco-2 cells treated with L. plantarum MB452. Describes the microarray analysis and qRT-PCR analysis. Include Table S4 showing the qRT-PCR primers. [file 1471-2180-10-316-S3.DOC]

**Additional file 3.** Analysis of gene expression of Caco-2 cells treated with *L. plantarum* MB452

**Microarray analysis**

The total RNA was extracted from the Caco-2 cells using TRIzol (Invitrogen, Auckland, New Zealand) according to the manufacturer’s instructions. The extracted RNA was then precipitated by the addition of 1 μL glycogen (5 mg/mL), 0.1 volumes 3M sodium acetate and 2.5 volumes 100% ethanol and incubated at -85°C for 30 minutes prior to centrifugation (12000g for 30 minutes). The pellet was washed with 100% ethanol and recollected (12000g for 20 minutes) three times. The RNA quantity and purity was determined using a Nanodrop ND-1000 spectrophotometer (Nanodrop Technologies, Wilmingtom, DE, USA), to ensure the samples had an OD_260nm_/OD_230nm_ ratio > 1.8. The extracted RNA was purified using RNeasy mini columns (QIAGEN, San Diego, CA, USA) and RNA quality was assessed using an RNA 6000 NanoLabChip kit with the Agilent 2100 Bioanalyser (Agilent Technologies, Palo Alto, CA, USA). For microarray analysis, all samples were required to have had an OD_260nm_/OD_280nm_ ratio, a Bioanalyser 28s/18s peak ratio > 1.5 and a RNA integrity number (RIN) > 8.

An equal amount of RNA from three wells of the same treatment was pooled together to yield enough RNA for the gene expression analysis (microarray and qRT-PCR); two control pools and two pools treated with *L. plantarum* MB452. Equal amounts of RNA from all 12 wells were pooled together to make the reference RNA sample. The RNA samples were labeled using the Agilent Low RNA Input Linear Amplification Kit (5183-3523). The total RNA (500 ng) was amplified and reverse transcribed to cDNA using T7-polymerase. The cDNA was labeled with either cyanine3 (reference sample) or cyaninine5 (experimental samples) labeled CTP dye (Perkin-Elmer/NEN Life Sciences, Boston, MA, USA). Dye incorporation was analyzed using a Nanodrop ND-1000 spectrophotometer to ensure all samples had a yield of > 825 ng of cRNA and specific activity of > 8 pmol dye/µg cRNA.

The amplified and fluorescently-labeled cRNA were hybridized using the Agilent oligonucleotide microarray *in situ* hybridization kit (5184-3568). Experimental and reference cRNA (750 ng of each) was mixed with the control target, fragmented for 30 minutes at 60°C in the dark and hybridized onto Agilent Technologies 44k whole human genome oligonucleotide arrays (G4112A) in a rotation oven at 60°C, at 4 rpm for 17 hours. Following hybridization the chambers were dissembled and the microarray slides were washed in Agilent Wash Solutions I, II and III, and air dried. The slides were scanned using a GenePix Professional 4200A scanner (Molecular Devices, Sunnyvale, CA, USA). Each slide was scanned at two photomultiplier tube (PMT) voltages intensities - a high scan of approximately 600 PMT, and a low scan of approximately 450 PMT. The values from these two scans were combined to give a single set of data using a modified version of the method of Lyng *et al* [1]. The high intensity scan values were used for all spots except those nearing saturation. For higher intensity spots, the low intensity scan values were substituted into the equation describing the linear relationship between the low and high scans and the resultant reading used. Spot identification and quantification was performed with GenePix 6.0 software (Molecular Devices). The images were also manually checked and any poor quality spots were removed before statistical analysis.

The Limma package in Bioconductor was used to analyze the microarray data [2]. No background correction was used as image analysis of the foreground and background fluorescence intensity measurements did not detect any relationship. The data were normalized using a loess smoothing procedure to remove systematic spatial effects, and internal Agilent control spot values (Pro25G and EQC) were removed before further analysis. For each spot, a modified t-test was calculated using an empirical Bayes approach [3]. Genes with a fold change greater than 1.2 and a modified p-value less than 0.05 were considered differentially expressed. Differentially expressed genes were clustered into functional groups and pathways using Ingenuity Pathway Analysis (IPA version 7.1; Ingenuity Systems Inc., Redwood City, CA, USA), Gene Ontology categories and KEGG pathways using EASE (version 2.0)[4].

**qRT-PCR analysis**

The expression of tight junction-related genes differentially expressed from the microarray analysis was confirmed using qRT-PCR. For this analysis, individual well samples, not pools (as in the microarray analysis), were used. All reagents (LightCycler 480 ProbesMaster), instruments (LightCycler 480) and software (Universal ProbeLibrary) were obtained from Roche Applied Sciences (Auckland, NZ), unless otherwise stated. 500 ng of total RNA isolated from CaCo-2 cells (from the microarray experiment) was treated with DNase I (Ambion DNA-free™ kit, Applied Biosystems, NZ) following the manufacturer's guidelines. The total RNA (purity 1.8–2.0) was reverse transcribed into cDNA with the Transcriptor First Strand cDNA Synthesis Kit (Roche Applied Science, NZ) using random hexamers according to the manufacturer's protocol. The cDNA was stored at -20°C prior to qRT-PCR.

The expression of seven target genes relative to three reference genes was assessed using the standard curve method. Primers and probes for all genes were designed using Universal ProbeLibrary software (Supplemental Table 1). The reference genes (GAPD, SDHA and YWHAZ) were chosen based on the findings of Vandesompele *et al* [5] and their log ratios in the microarray data (close to 1; not differentially expressed). Five target genes (ZO-1, ZO-2, OCLN, CGN and ACTB) were chosen from the tight junction-related genes that were differentially expressed (all up-regulated) in the microarray analysis. The two other target genes, GJA7 and CLDN3, were chosen to be included because they were down-regulated and not differentially expressed, respectively, in the microarray analysis.

A serial dilution of cDNA (1:10, 1:50, 1:100, 1:500 and 1:1000) from each treatment was used to generate standard curves for all genes. A 1:25 dilution of cDNA from each treatment was used to determine the expression. Appropriate no-template and genomic DNA controls were used. All reactions were prepared in duplicate and included 5.0 µL cDNA, 3.8 µL genomic-grade water, 0.4 µL probe (10 µM), 0.4 µL forward primer (20 µM), 0.4 µL reverse primer (20 µM), 20 µL LightCycler 480 ProbesMaster mix in a 96 well optical plate and covered with an optical adhesive cover. The PCR thermal cycling conditions were; 95°C for 10 seconds, 45 cycles of 95°C for 10 seconds, 60°C for 30 seconds and 72°C for 1 second, and finally 40°C for 10 seconds. The data was analyzed using Relative Expression Software Tool (REST 2008) version 2.0.7 with efficiency correction [6].

1. Lyng H, Badiee A, Svendsrud DH, Hovig E, Myklebost O, Stokke T: **Profound influence of microarray scanner characteristics on gene expression ratios: analysis and procedure for correction**. *BMC Genomics* 2004, **5**(1):10.

2. Smyth G: **Limma: linear models for microarray data**. In: *Bioinformatics and Computational Biology Solutions using R and Bioconductor.* Edited by Gentleman R, Carey V, Dudoit S, Irizarry R, Huber W. New York: Springer; 2005: 397–420

3. Smyth GK: **Linear Models and Empirical Bayes Methods for Assessing Differential Expression in Microarray Experiments**. *Statistical Applications in Genetics and Molecular Biology* 2004, **3**(1):Article 3.

4. Hosack DA, Dennis G, Jr., Sherman BT, Lane HC, Lempicki RA: **Identifying biological themes within lists of genes with EASE**. *Genome Biol* 2003, **4**(10):R70.

5. Vandesompele J, De Preter K, Pattyn F, Poppe B, Van Roy N, De Paepe A, Speleman F: **Accurate normalization of real-time quantitative RT-PCR data by geometric averaging of multiple internal control genes**. *Genome Biol* 2002, **3**(7):34.

6. Pfaffl MW, Horgan GW, Dempfle L: **Relative expression software tool (REST) for group-wise comparison and statistical analysis of relative expression results in real-time PCR**. *Nucleic Acids Res* 2002, **30**(9):e36.

**Table S4.** Primers and probes used for qRT-PCR designed using Universal ProbeLibrary software (www.roche-applied-science.com/sis/rtpcr/upl/index.jsp).

| **Gene** | **Gene ID** | **Primer/probe ^a^** | **Sequence** |
| --- | --- | --- | --- |
| GAPD ^b^ | NM_002046.3 | Forward | agccacatcgctcagacac |
|  |  | Reverse | gcccaatacgaccaaatcc |
|  |  | Probe | TGGGGAAG (60) |
|  |  |  |  |
| SDHA ^b^ | NM_004168.1 | Forward | agaagccctttgaggagca |
|  |  | Reverse | cgattacgggtctatattccaga |
|  |  | Probe | GGAGGAAG (69) |
|  |  |  |  |
| YWHAZ ^b^ | NM_003406.2 | Forward | cgttacttggctgaggttgc |
|  |  | Reverse | tgcttgttgtgactgatcgac |
|  |  | Probe | TGGTGATG (9) |
|  |  |  |  |
| GJA7 ^c^ | NM_005497 | Forward | cccaggctcaagcaatca |
|  |  | Reverse | aacagaattagccgagtatggtg |
|  |  | Probe | AGCTGGA (33) |
|  |  |  |  |
| ZO-1 ^c^ | NM_003257.3 | Forward | cagagccttctgatcattcca |
|  |  | Reverse | catctctactccggagactgc |
|  |  | Probe | CAGCAGCC (66) |
|  |  |  |  |
| ZO-2 ^c^ | NM_004817.2 | Forward | ggccaggttacagagaatgc |
|  |  | Reverse | gcttctgggcaatttcgat |
|  |  | Probe | GCTCCAGG (1) |
|  |  |  |  |
| CLDN3 ^c^ | NM_001306 | Forward | ctacgaccgcaaggactacg |
|  |  | Reverse | gtggtggtgttggtggtg |
|  |  | Probe | CCACCACC (77) |
|  |  |  |  |
| CGN ^C^ | NM_020770.1 | Forward | gcaagatgcaacccagga |
|  |  | Reverse | agtctcctccagctccctct |
|  |  | Probe | GGCAGAAG (29) |
|  |  |  |  |
| OCLN ^c^ | NM_002538.2 | Forward | aggaaccgagagccaggt |
|  |  | Reverse | caatgccctttagcttccaa |
|  |  | Probe | GCAGCAAA (84) |
|  |  |  |  |
| ACTB ^c^ | NM_001101.2 | Forward | ccaaccgcgagaagatga |
|  |  | Reverse | ccagaggcgtacagggatag |
|  |  | Probe | CCAGGCTG (64) |

^a^ probe number refers to Roche UPL code

^b^ reference gene

^c^ target gene
